# Supplementary material for: 6 nm super-resolution optical transmission and scattering spectroscopic imaging of carbon nanotubes using a nanometer-scale white light source
Source: Nat Commun. 2021 Nov 25;12:6868. doi: 10.1038/s41467-021-27216-5 (PMC8617169; doi:10.1038/s41467-021-27216-5)
Supplement: Supplementary file 1 — Supplementary Information [file 41467_2021_27216_MOESM1_ESM.pdf]

6 nm super-resolution optical transmission and scattering spectroscopic imaging of carbon nanotubes using a nanometer-scale white light source

Xuezhi Ma[1], Qiushi Liu[1], Ning Yu[2], Da Xu[1], Sanggon Kim[2], Zebin Liu[3], Kaili Jiang[3], Bryan M. Wong[2,4], Ruoxue Yan[2,4*] and Ming Liu[1,4*]

Addresses:

[1] Department of Electrical and Computer Engineering, University of California - Riverside, Riverside, California 92521, United States

[2] Department of Chemical and Environmental Engineering, University of California - Riverside, Riverside, California 92521, United States

[3] State Key Laboratory of Low Dimensional Quantum Physics and Department of Physics, Tsinghua University, Beijing, 100084, China

[4] Materials Science and Engineering program, University of California - Riverside, Riverside, California 92521, United States

Corresponding author information: mingliu@ucr.edu, rxyan@engr.ucr.edu

Table of Contents

1. SW-CNT sample preparation 2
2. ROI selection in the spectrometer camera 3
3. Polarization-resolved Fourier-Transform (*k*-space) imaging 4
4. The influence of *k*-space filter size 5
5. The spatial resolution of the transmission and scattering spectroscopic images ... 6
6. AFM nanomanipulation for strained SWNTs 7
7. AFM and Raman characterizations for the SWNT samples 9
8. Far-field radiation and its probe-sample-distance dependence 10
9. References: 12

1. SW-CNT sample preparation

We use the aligned SWCNTs provided following the CVD synthesis method described in a prior work[1]. The aligned SWCNT arrays were grown on a quartz crystal substrate and then transferred onto a thinner quartz substrate by a standard PMMA-assisted wet transfer method. We transferred the CNTs array onto the glass with gold markers (See Supplementary Figure 1), which allows us to put SWCNTs along a specific direction for scanning (e.g. x-direction in Figure 1). With the SWCNT orientation fixed, we can define two regions of interest in the spectrometer camera.

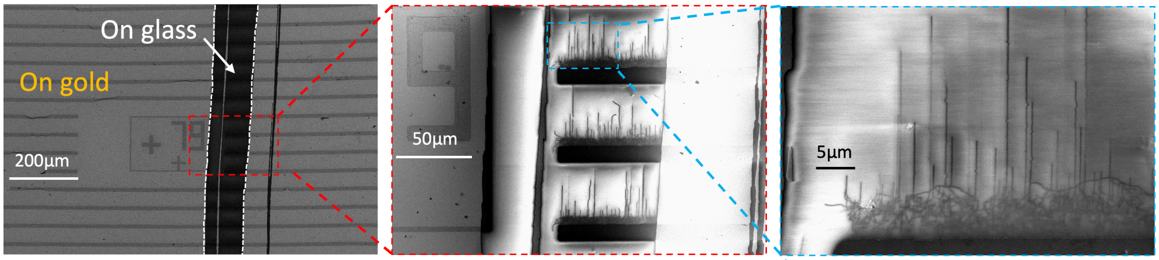

Supplementary Figure 1. SEM images of aligned-CNT array transferred on glass substrate with gold marker.

2. ROI selection in the spectrometer camera

Supplementary Figure 2shows scanning probe images, acquired from the side camera port of the inverted optical microscope that is integrated with the NSOM scanning module (Nanonics Multiview 2000). Supplementary Figure 2a is a high-resolution image recorded by a color CCD, showing a high-quality doughnut shape with a radial polarization (Supplementary Figure 3). Supplementary Figure 2b is the image of the same probe acquired by a spectrometer camera (Princeton Instrument Acton 2300), with the grating set at zero-order reflection mode. The white dashed lines in Supplementary Figure 2a indicate the location of the front slit, which cut the doughnut ring into half to improve the spectral resolution; otherwise, the ring can broaden the peaks in the spectrum (by a few pixels). The real slit size in Supplementary Figure 2b is around 100 μm, so the spectral resolution is not compromised.

The doughnut pattern occupies about 7 horizontal lines (7 pixels along the vertical direction) in our spectrometer CCD camera. We group them into two regions of interest, labeled as $ROI_{\parallel}$ and $ROI_{\perp}$. We could do this grouping because we already have the SWCNT fixed along the x-axis (corresponding to the vertical direction in the spectrometer reference frame). We set only two groups because the usb2.0 cable used by the spectrometer has a low data transfer rate, as a 2×1400 data array already consumed around 1.5 seconds, even though our exposure time was only 0.3 seconds.

With that being said, in the ideal case (Supplementary Figure 2c), the ROIs can be divided finer if the spectrometer CCD has a smaller pixel size and higher data transfer rate. With more

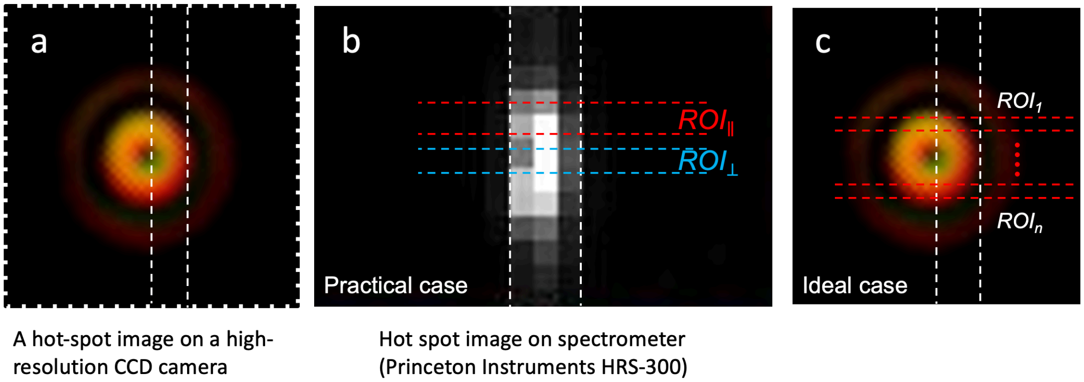

Supplementary Figure 2. Probe tip images acquired by **a,** a high-resolution CCD camera and **b,** spectrometer camera (Princeton Instruments Acton 2300), respectively. The entrance slit of the spectrometer is marked by white dashed lines. **c,** Ideally, more ROIs can be picked for a better polarization analysis.

ROIs defined, the predetermination of sample orientations is no longer required.

3. Polarization-resolved Fourier-Transform (*k*-space) imaging

As shown by the *k*-space measurement in Fig. 1 in the manuscript, a large-diameter circle emerges when the AgNW probe approaches the substrate. This circle represents the 2nd-order lobe in the far-field radiation pattern, which originates from the interaction between the superfocused TM_0 mode at the AgNW tip apex and its image hotspot in the sample/substrate. Since both the TM_0 mode and its image are azimuthally symmetrical, the far-field pattern also has azimuthal symmetry with radial polarization.

To confirm this radial polarization, we performed the *k*-space measurement on a setup sketched in Supplementary Figure 3a. In the measurement, a AgNW probe attached to a tapping-mode tuning fork was launched onto a glass slide substrate to generate the far-field radiation, which was collected by an oil-immersion lens (100 ×, NA=1.3), analyzed by a linear polarizer analyzer (LP), and imaged by two lenses (L1 and L2) onto a CCD camera. The polarizer analyzer was placed before the beam splitter (BS) to avoid the reflection process's depolarization effect. During the experiment, the probe oscillation peak-to-peak amplitude was retained at around 10 nm to maintain a strong 2nd-order lobe component.

Supplementary Figure 3b illustrates a typical "white"-color *k*-space pattern with the probe launched. The excitation light was coupled from a tungsten-halogen lamp (SLS201L, Thorlabs) into single-mode fiber (SM600) via a fiber collimator (F230FC-B, Thorlabs). The light source's nominal color temperature is 2796 K (~ 1000nm), which leads to the red-color scheme of the *k*-space images. The *k*-space image contains two regions: a circular rainbow region in the middle that comes from the direct far-field radiation of the superfocused TM_0 mode, and a wide ribbon outside of the dashed circle in Supplementary Figure 3b that originates from the 2nd-order lobe far-field radiation. Polarization analyzation (Supplementary Figure 3c~f) confirms that both the central TM_0 part and the 2nd-lobe part are radially polarized.

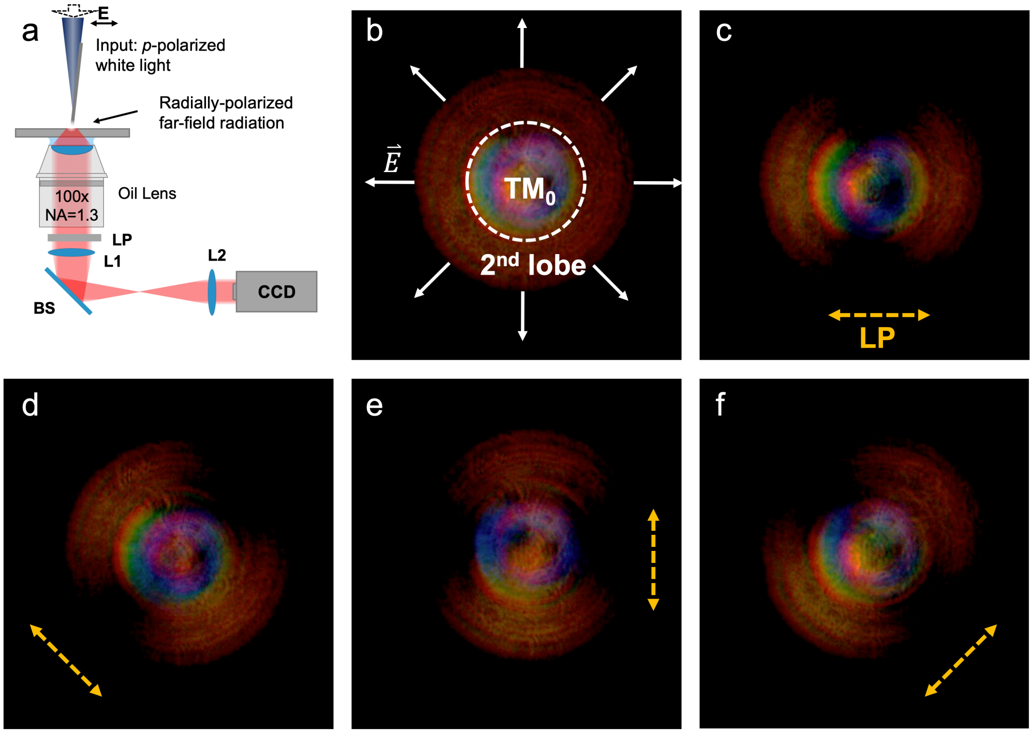

Supplementary Figure 3. a, A schematic diagram of the experiment setup for *k*-space polarization measurement. The incident light (*p*-polarization) from a tungsten-halogen lamp was coupled into the AgNW probe, which was launched onto a glass substrate via tapping-mode tuning fork control feedback. The far-field radiation was collected by an oil-immersion lens, analyzed by a linear polarizer analyzer (LP), and imaged by two lens (L1, L2) onto a CCD camera. **b**, a typical *k*-space image without a linear polarizer. **c**~**f**, polarization analysis. Yellow dashed arrows indicate the transmission axis of the LP.

4. The influence of *k*-space filter size

The (18, 16) SW-CNT are examined by two *k*-space filters, with NA = 0.7 and 0.9. Compared with the results acquired with the 0.7 NA filter, the 0.9 NA filter gives a better image quality for the absorption images ($ROI_{\parallel}$) but cannot provide any scattering signal ($ROI_{\perp}$). This phenomenon can be explained by the reasons below:

1) Unlike absorption, the CNT scatters the light to a narrow solid angle, and the scattering signal can only be detected within a small range of *k*-space. At NA > 0.9, the scattering signal hardly exists. This radiation pattern might differ from the doughnut-shaped dipole radiation pattern (the parallel excitons can be considered as an electric dipole, with its polarization along the CNT axis[2]). We suspect the lack of high-NA information should come from the influence of the silver tip, which may serve as the reflector in a Yagi-Uda antenna.
2) The absorption images seem to show a better signal-to-noise ratio, which may come from the fact that high-NA signals have less influence from other noise, such as the Mie scattering from sample contaminations.

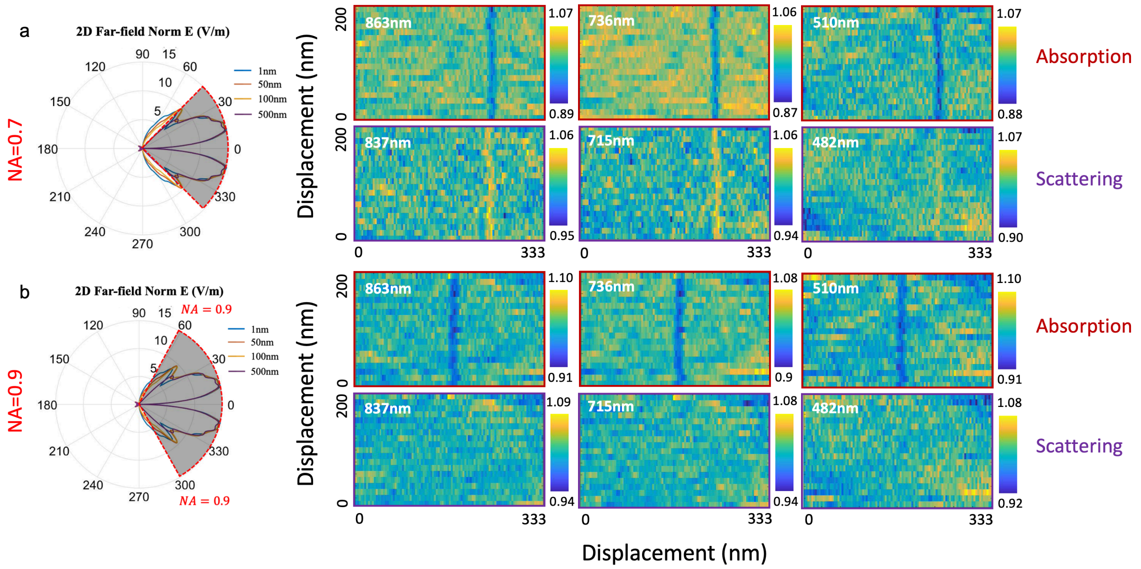

Supplementary Figure 4. Comparison of the scanning images on an (18,16) CNT with **a,** a smaller *k*-space filter (NA = 0.7) and **b,** a larger *k*-space filter (NA = 0.9).

5. The spatial resolution of the transmission and scattering spectroscopic images

We use the 2D Gaussian fitting to determine the spatial resolution. In the full-color CNT mapping, we continuously picked out a series of spectra that perpendicularly across the (18, 16) SW-CNT, as shown in Supplementary Figure 5. We performed a 2D Gaussian fitting (in both the displacement and wavelength direction) and found the spatial resolution at **6 nm** for the short-

wavelength peak/valley at 510 nm.

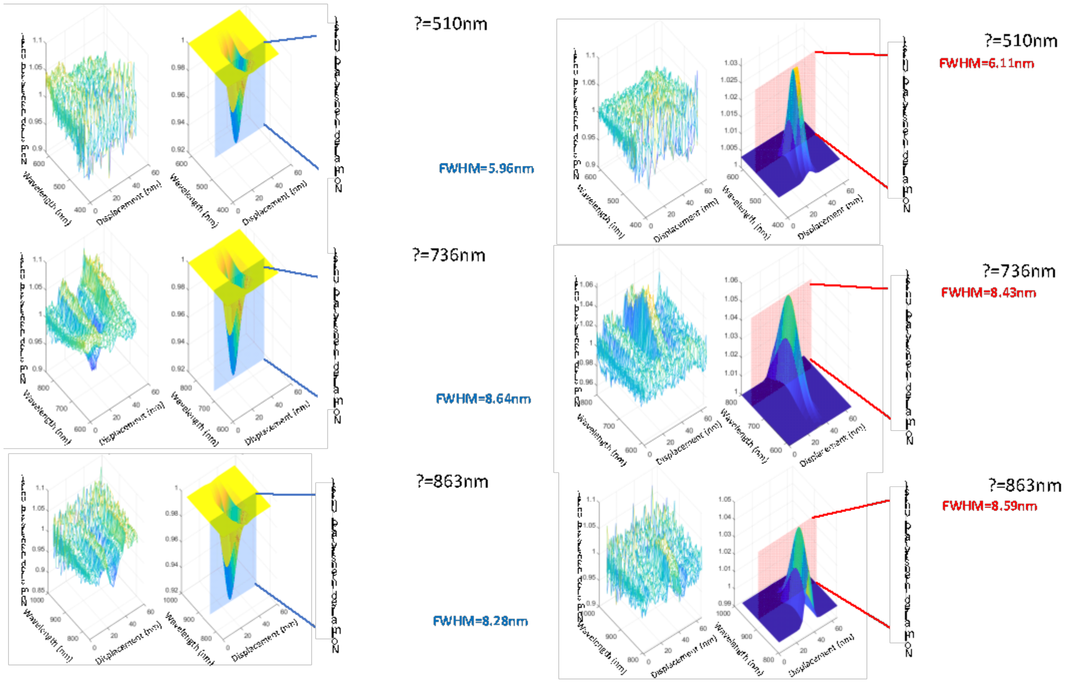

Supplementary Figure 5. 2D-Gaussian fitting for the spatial resolution calculation. **a~c**, absorption spectra. **d~f**, scattering spectra. This (18, 16) SWCNT is the same one used in Figure 2. The spatial resolution is 6 nm for the short-wavelength valley/peak in **a** and **d**.

6. AFM nanomanipulation for strained SWNTs

Contact-mode AFM control (SmartSPM, Horiba) was employed to move a cantilever probe (OMCL-AC160TS-R3, Olympus) on the sample surface to manipulate the SWNT, where the contact force was adjusted by changing the setpoint of the feedback. Supplementary Figure 6a~c illustrate schematics of the manipulation process. After locating the position of the target SWNT, the AFM probe was programmed to scan across the nanowire at approximately 100 nm/s. The cantilever's pressing force on the quartz substrate was carefully adjusted to around 100 nN to realize the manipulation while avoiding any potential damage to the target SWNT. When the probe started to drag the SWNT from the contact point a (Supplementary Figure 6b), uniaxial strains were created in the triangle region (segment ab and ac in Supplementary Figure 6e). When the resultant force (tension from the two SWNT segments and the friction from the substrate) became

larger than the maximum pressure affordable from the AFM probe to the SWNT, the SWNT was detached from the AFM probe (Supplementary Figure 6c). At this moment, the force from the stored strain in the SWNT was usually larger than the maximum friction force from the quartz substrate, leading to the sliding of the SWNT on the latter. Since point *a* received the pulling forces from both segment *ab* and *ac*, it underwent a large restoring force than the other part of the SWNT and was the first to slide. Therefore, the strain in point *a* is primarily reduced after the relaxation process. After the relaxation process, the bent SWNT has a crescent shape, as observed in Figure 4c and d in the manuscript. Supplementary Figure 6g and h show another example of before and after the bending process.

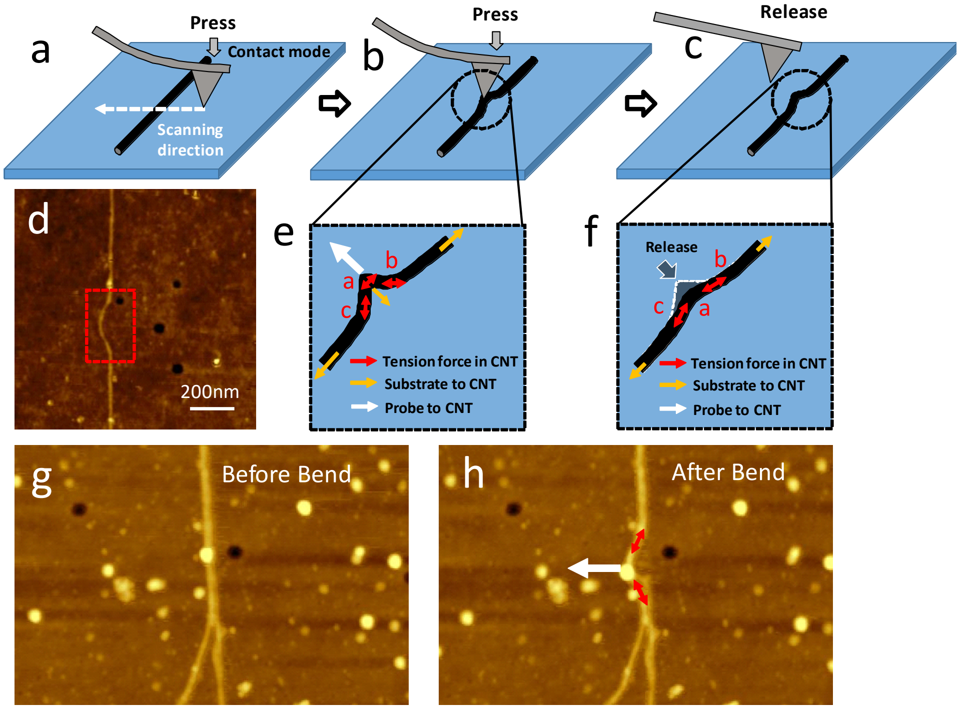

Supplementary Figure 6. a-c, Schematic of bending a SWNT using the contact mode AFM nanomanipulation method. **d,** the bend SWNT used in the manuscript. **e** and **f,** zoomed-in schematics illustrate the force analysis on the SWNT during the bending process. The red arrows show the tension force stored inside the SWNT. The yellow arrows show the friction force between the substrate and the SWNT. The white arrow shows the force applied by the cantilever probe tip. **g** and **h,** the AFM images show an example of before and after a bending manipulation.

7. AFM and Raman characterizations for the SWNT samples

Complementary characterizations on the SWNT samples include tapping-mode AFM measurement and Raman characterizations. The tapping mode AFM measurement can provide topographical information with nanometer-level lateral accuracy and sub-nanometer-level vertical accuracy [3,4]. Supplementary Figure 7a, d, and g show the tapping mode AFM height images of the three different SWNTs used for the hyperspectral imaging measurement in the manuscript. Supplementary Figure 7b, e, and h are the line profiles from the marked regions in a, d, and g, which give heights around 2.3 nm, 1.0 nm, and 1.4 nm, respectively. These measured results are close to the theoretical predictions: 2.339 nm for (18, 16), 0.966 nm for (8, 6), and 1.384 nm for (12, 8) [5]. (Supplementary Table 1)

The radial-breathing mode (RBM) of a SWNT is also sensitive to the SWNT geometry and has been widely used as an accurate gauge to determine diameters. The Raman spectra of the three SWNTs are shown in Supplementary Figure 7c, f and i, and their diameters are determined to be 2.33 nm, 0.97 nm, and 1.38 nm, following the equation [6,7]:

$$\omega_{RBM}(cm^{-1}) = \frac{217.8}{d(nm)} + 15.7 \quad (1)$$

Supplementary Table 1. Comparison of the diameters (Unit: nm)

SWNT type	Modeling	AFM	Raman
(18, 16)	2.339	2.3	2.33
(8, 6)	0.966	1.0	0.97
(12, 8)	1.384	1.4	1.38

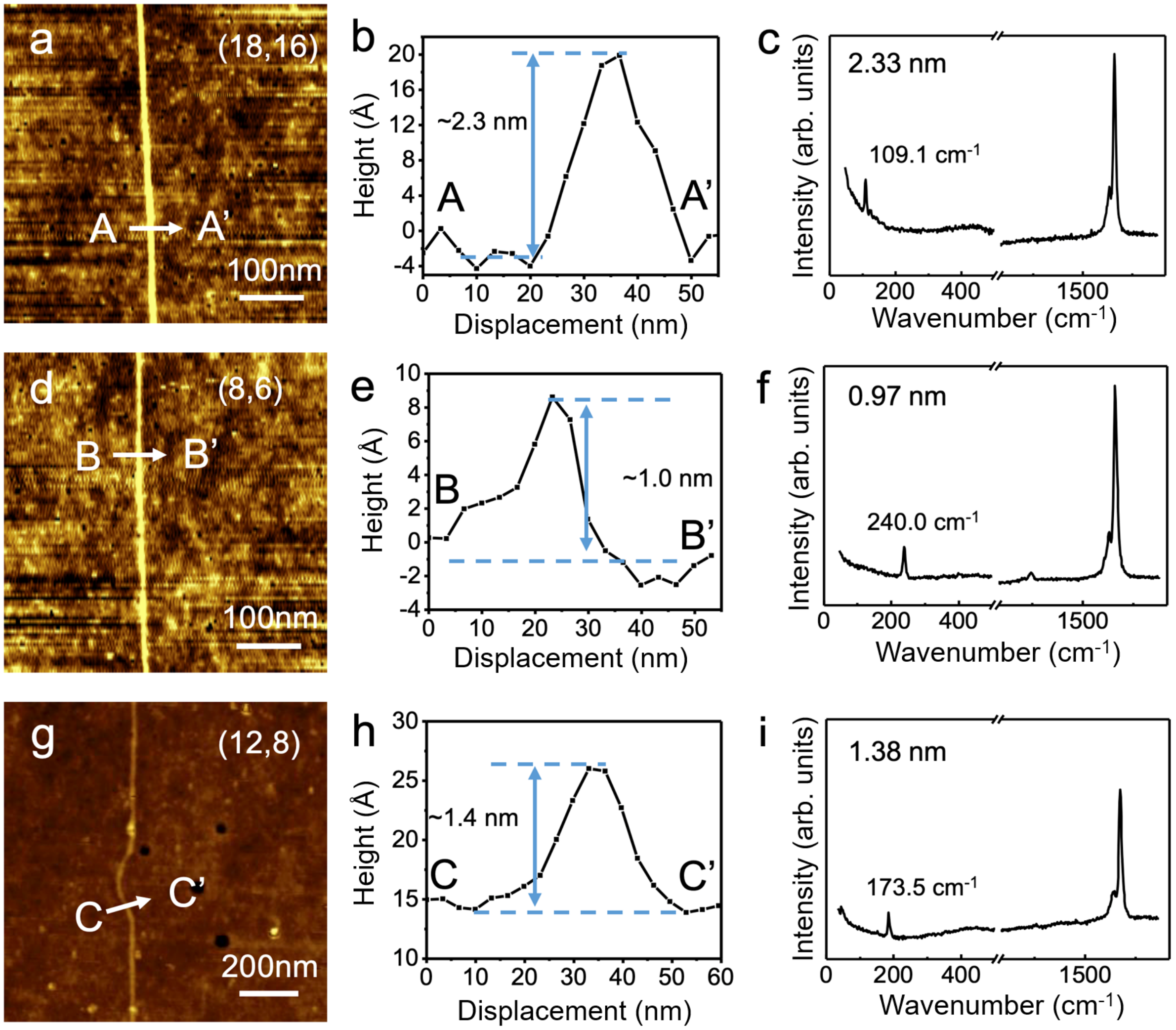

Supplementary Figure 7. a, d and **g,** Tapping-mode AFM topographic images of three different SWNTs. **b, e** and **h**, The line profiles from the marked regions. **c, f** and **i,** corresponding Raman spectra collected by a micro-Raman system (LabRam, Horiba, λ = 532 nm). The RBM peak locations and the calculated diameters using eq. (1) are labeled inside the figures.

8. Far-field radiation and its probe-sample-distance dependence

3D full-wave simulations were performed using commercial software (COMSOL Multiphysics) to analyze the far-field radiation and its reliance on the probe-sample distance. The 3D modeling contained a sharp-tip silver nanowire (200 nm in diameter, 45° in taper angle, and 5 nm in tip radius) placed perpendicular to a glass substrate to imitate the experiment conditions. The incident light (λ = 750 nm) was launched on the flat end of the AgNW in the form of surface

plasmon polariton (TM_0 mode), and its far-field radiation emitted by the sharp-tip and refracted by the glass substrate is used for data analysis. As shown in the inset of Supplementary Figure 8a, the far-field pattern contains two major lobes (maximum at ~ ±15°), which are from the projection of the circular far-field radiation on the E-plane. As the probe approaches the glass substrate ($z \rightarrow 0$ nm), two side-lobes (2nd-order lobe) emerge, with their maximum peaks at around ±45°. These lobes originate from the interference between the TM_0 mode at AgNW tip and its image in the glass substrate. The First Null Beam Width (FNBW, red dashed lines) is around 68° (±34° in Supplementary Figure 8b), which corresponds to a NA of approximately 0.56.

The *k*-space filter used in the experiment was about 0.7 NA (~ ±44.5°), which equivalents to discarding the signals from -44.5° to 44.5° in Supplementary Figure 8b (shadowed region). The radiation power from the high-*k* components outside of the shaded area is sensitive to the probe-sample distance *z*, as shown in Supplementary Figure 8c. The fitting curve indicates that the radiation intensity drops by half at around *z* = 30 nm, which agrees with the trend shown by the *k*-space images (Supplementary Figure 8d~g).

It is worth noting that in the experiment, the intensity-z curve is steeper than the simulated

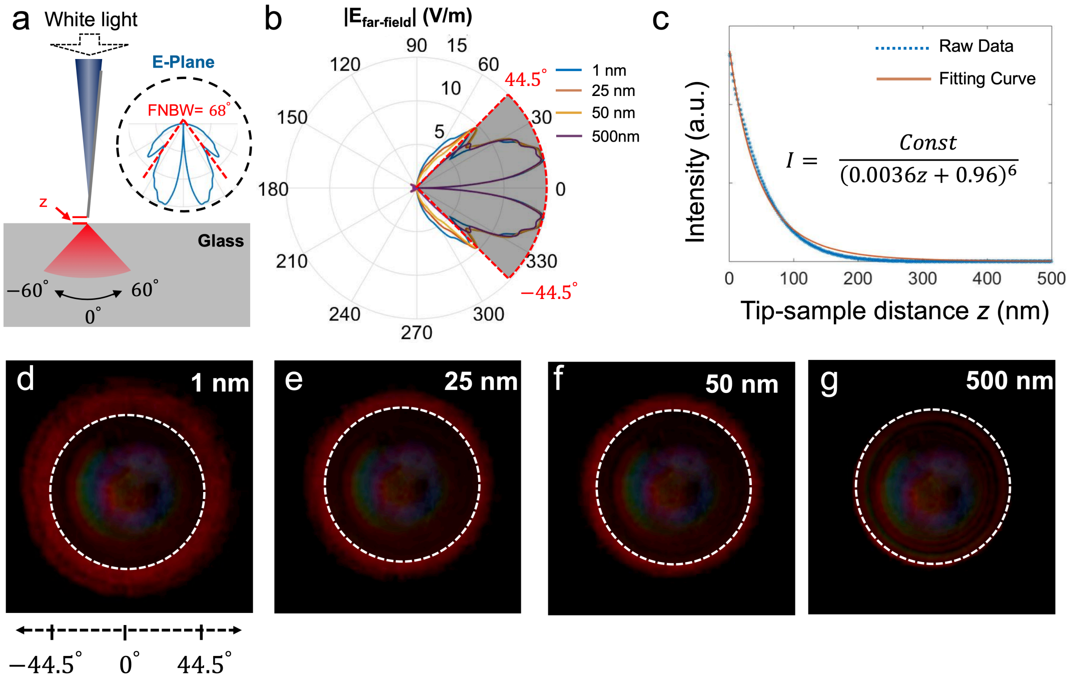

Supplementary Figure 8. a. The configuration for the numerical simulation of the far-field distribution. The first-null beam-width (FNBW) of the major lobe is 68°. **b.** The 2D distribution of the far-field E component with different tip-sample distances. The shadowed region indicates the range of a *k*-space filter with NA= 0.7. **c.** The total transmission intensity of the light outside of the shadowed region versus different tip-sample distance *z*. **d-g.** *k*-space images for different tip-sample distances.

result in Supplementary Figure 8c, because the shift of the probe (with larger *z*) can shrink the overall size of its *k*-space pattern and thus get more blocked by the *k*-space filter, an effect similar to a confocal imaging system. Therefore, the sensitivity of far-field intensity over the tip-sample distance is enhanced.

9. References:

1 Wang, J. *et al.* Growing highly pure semiconducting carbon nanotubes by electrotwisting the helicity. *Nature Catalysis* **1**, 326-331, doi:10.1038/s41929-018-0057-x (2018).

2 Blancon, J.-C. *et al.* Direct measurement of the absolute absorption spectrum of individual semiconducting single-wall carbon nanotubes. *Nature Communications* **4**, 2542, doi:10.1038/ncomms3542 (2013).

3 Tortonese, M., Barrett, R. & Quate, C. Atomic resolution with an atomic force microscope using piezoresistive detection. *Applied physics letters* **62**, 834-836 (1993).

4 Ma, X. *et al.* Sharp-tip silver nanowires mounted on cantilevers for high-aspect-ratio high-resolution imaging. *Nano letters* **16**, 6896-6902 (2016).

5 Popov, V. N. Curvature effects on the structural, electronic and optical properties of isolated single-walled carbon nanotubes within a symmetry-adapted non-orthogonal tight-binding model. *New Journal of Physics* **6**, 17 (2004).

6 Araujo, P. T. *et al.* Third and fourth optical transitions in semiconducting carbon nanotubes. *Physical Review Letters* **98**, 067401 (2007).

7 Takase, M. *et al.* Selection-rule breakdown in plasmon-induced electronic excitation of an isolated single-walled carbon nanotube. *Nature Photonics* **7**, 550 (2013).
